# Prediction of venetoclax activity in precursor B-ALL by functional assessment of apoptosis signaling

**DOI:** 10.1038/s41419-019-1801-0

**Published:** 2019-07-29

**Authors:** Felix Seyfried, Salih Demir, Rebecca Louise Hörl, Felix Uli Stirnweiß, Jeremy Ryan, Annika Scheffold, Mariana Villalobos-Ortiz, Elena Boldrin, Julia Zinngrebe, Stefanie Enzenmüller, Silvia Jenni, Yi-Chien Tsai, Beat Bornhauser, Axel Fürstberger, Johann Michael Kraus, Hans Armin Kestler, Jean-Pierre Bourquin, Stephan Stilgenbauer, Anthony Letai, Klaus-Michael Debatin, Lüder Hinrich Meyer

**Affiliations:** 1 Department of Pediatrics and Adolescent Medicine, Ulm University Medical Center, Ulm, Germany; 2 International Graduate School of Molecular Medicine, Ulm University, Ulm, Germany; 3 Department of Medical Oncology, Dana-Farber Cancer Institute, Boston, MA USA; 4 Department of Internal Medicine III, Ulm University Medical Center, Ulm, Germany; 5 Department of Oncology and Children’s Research Center, University Children’s Hospital Zurich, Zurich, Switzerland; 6 Institute of Medical Systems Biology, Ulm University, Ulm, Germany

**Keywords:** Acute lymphocytic leukaemia, Apoptosis, Acute lymphocytic leukaemia, Paediatric cancer

## Abstract

Deregulated cell death pathways contribute to leukemogenesis and treatment failure in B-cell precursor acute lymphoblastic leukemia (BCP-ALL). Intrinsic apoptosis signaling is regulated by different proapoptotic and antiapoptotic molecules: proapoptotic BCL-2 homology domain 3 (BH3) proteins activate prodeath molecules leading to cellular death, while antiapoptotic molecules including B-cell lymphoma 2 (BCL-2) prevent activation of prodeath proteins and counter-regulate apoptosis induction. Inhibition of these antiapoptotic regulators has become a promising strategy for anticancer treatment, but variable anticancer activities in different malignancies indicate the need for upfront identification of responsive patients. Here, we investigated the activity of the BCL-2 inhibitor venetoclax (VEN, ABT-199) in B-cell precursor acute lymphoblastic leukemia and found heterogeneous sensitivities in BCP-ALL cell lines and in a series of patient-derived primografts. To identify parameters of sensitivity and resistance, we evaluated genetic aberrations, gene-expression profiles, expression levels of apoptosis regulators, and functional apoptosis parameters analyzed by mitochondrial profiling using recombinant BH3-like peptides. Importantly, ex vivo VEN sensitivity was most accurately associated with functional BCL-2 dependence detected by BH3 profiling. Modeling clinical application of VEN in a preclinical trial in a set of individual ALL primografts, we identified that leukemia-free survival of VEN treated mice was precisely determined by functional BCL-2 dependence. Moreover, the predictive value of ex vivo measured functional BCL-2 dependence for preclinical in vivo VEN response was confirmed in an independent set of primograft ALL including T- and high risk-ALL. Thus, integrative analysis of the apoptosis signaling indicating mitochondrial addiction to BCL-2 accurately predicts antileukemia activity of VEN, robustly identifies VEN-responsive patients, and provides information for stratification and clinical guidance in future clinical applications of VEN in patients with ALL.

## Introduction

Although survival rates of pediatric B-cell precursor acute lymphoblastic leukemia (BCP-ALL) patients have improved during the past decades, therapy-related toxicity and relapse occur in 10–20% of patients and are associated with a poor outcome, clearly emphasizing the need for novel, targeted treatment strategies^1,2^. Deregulation of survival and cell death pathways are hallmarks of cancer^3^, particularly of BCP-ALL^4,5^, and contribute to treatment failure and disease reoccurrence. B-cell lymphoma 2 (BCL-2) family members are key regulators of apoptosis and frequently overexpressed in lymphoid malignancies^6,7^. Proapoptotic BCL-2 homology domain 3 (BH3)-only proteins bind to the BH3-binding domain of antiapoptotic BCL-2 family members, activating intrinsic apoptosis signaling^8^. Small molecule inhibitors like ABT-737 and the orally bioavailable navitoclax (ABT-263) mimic the binding of BH3-only proteins to the BH-3 domains of antiapoptotic BCL-2, BCL-XL and BCL-W, thereby inducing apoptosis^9,10^. Navitoclax has shown antitumor activity in BCL-2-dependent malignancies, however co-occurring thrombocytopenia has limited its clinical application^11^. Venetoclax (VEN, ABT-199) is a BH3-mimetic molecule selectively targeting BCL-2 at low sub-nanomolar binding affinity, sparing BCL-XL and BCL-W therefore not affecting platelets^12^. Upon binding of VEN to BCL-2, proapoptotic proteins such as BIM are released activating downstream apoptosis signaling. However, cancer cells might sequester BIM and antagonize VEN-induced apoptosis^13^.

VEN showed high activity in patients with relapsed, 17p-deleted chronic lymphocytic leukemia (CLL), leading to drug-approval for these patients^11,14^. In addition, VEN showed clinical activity in other hematological malignancies, including blastic plasmacytoid dendritic cell neoplasm^15^, non-Hodgkin lymphoma^16^, acute myeloid leukemia^17^, and early T-cell precursor ALL^18^. In BCP-ALL, single-agent activity has been described in different subtypes in cell line, patient and patient-derived xenograft samples^19–22^. However, clinical application of VEN in BCP-ALL has just started evaluating the effects in patients with relapsed or refractory disease (ClinicalTrials.gov)^23,24^. Hence, clinical response data and consequently information on potential markers reflecting VEN sensitivity of individual patients will only be available after some time. Nevertheless, reliable upfront identification of patients who would potentially benefit from strategies targeting BCL-2 is mandatory for decision-making and therapy guidance of individual patients in future evaluation and clinical application of VEN in ALL patients.

In this study, we investigated VEN activity in a series of B-ALL cell lines and patient-derived xenografts and analyzed different leukemia characteristics with respect to VEN sensitivity. We identified that functional BCL-2 dependence of apoptosis signaling in ALL cells was the best marker associated with VEN response analyzed in isolated ALL cells ex vivo and, importantly, predicted VEN activity in a preclinical in vivo trial in xenografted mice.

## Results

### Heterogeneous VEN sensitivity in BCP-ALL

**Fig. 1:**
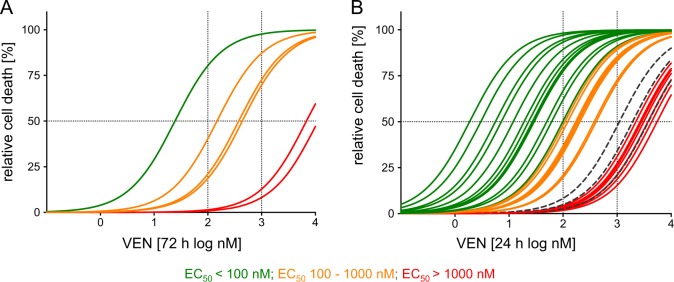
Heterogeneous sensitivities for VEN in BCP-ALL. Cell death induction (flow cytometry, forward/side scatter criteria) upon exposure of **a** BCP-ALL cell lines (*N* = 6, 72 h; from left [low EC_50_, sensitive] to right [high EC_50_, insensitive]: RS4;11, KOPN-8, UoCB6, REH, RCH-ACV, and Nalm-6), **b** patient-derived BCP-ALL xenograft samples (*N* = 27, 24 h, from left [low EC_50_, sensitive] to right [high EC_50_, insensitive]: PDX1, PDX2, …, PDX27) or peripheral blood mononuclear cells from healthy donors (*N* = 3, dashed lines) to increasing concentrations of VEN (0.1, 1, 10, 50, 100, 250, 500 nM, 1, 3, 5, and 10 µM) showing heterogeneous half maximal effective concentrations (EC_50_) indicating variable VEN sensitivities of BCP-ALL. (See also Supplementary Tables 1 and 2)

First, we investigated activity of the selective BCL-2 inhibitor VEN in a set of different BCP-ALL cell lines (*N* = 6) and patient-derived xenografts (PDXs, *N* = 27). Analysis of cellular viability after exposure to increasing concentrations of VEN showed that four out of six cell lines investigated were sensitive to BCL-2-inhibition with half maximal effective concentrations (EC_50_) in the nanomolar range (RS4;11: 24, KOPN-8: 148, UoCB6: 376, REH: 438 nM), while the two other lines showed clearly higher EC_50_ values at micromolar concentrations indicating VEN insensitivity (RCH-ACV: 6.8, NALM-6: 11.3 µM) (Fig. 1a, Supplementary Table 1).

Next, we analyzed VEN sensitivities of all together 27 patient-derived xenograft leukemia samples. Twenty (74%) showed cell death in response to VEN at nanomolar concentrations with EC_50_ values ranging from 1.8 to 410 nM in contrast to insensitivity in seven samples (26%; EC_50_ above 1 µM ranging from 2.2 to 5.5 µM) (Fig. 1b, Supplementary Table 2). VEN responses were reevaluated in two individual leukemias obtained from three consecutively transplanted recipients showing the same drug sensitivities confirming stability of drug sensitivity upon serial re-transplantation in the model (Supplementary Fig. 1). Moreover, we also addressed activity on healthy white blood cells investigating peripheral blood mononuclear cells obtained from three healthy donors, which showed only minimal responses at high VEN concentrations corresponding to EC_50_ values of insensitive leukemias (1.1, 1.9, and 3.8 µM) (Fig. 1b). Thus, VEN shows antileukemia activity in the majority of BCP-ALL samples; however, sensitivities vary including a subset of leukemia samples showing insensitivity.

### VEN sensitivity is independent of recurrent genetic alterations and leukemia characteristics

Given the heterogeneity of VEN responses observed in individual leukemias and the diversity of precursor B-ALL with different genetic alterations relevant for patient outcome and treatment stratification^25,26^, we addressed whether specific leukemia characteristics including genetic aberrations recurrently found in BCP-ALL are associated with VEN sensitivity (Supplementary Fig. 2). Immunophenotypes and patient gender were distributed independently of VEN sensitivities, and similar VEN sensitivities were seen in leukemias from patients with or without relapse (*U*-test, *P* = 0.341) and no differences in patient age were observed between VEN sensitive and insensitive ALL samples (*U*-test, *P* = 0.464) (Supplementary Fig. 2A). Four of the leukemia samples were derived from infants below one year of age and characterized by a pro-B ALL immunophenotype and either *MLL*/*AF4* (*N* = 1) or *MLL*/*ENL* (*N* = 3) gene fusions. The *MLL*/*AF4*-positive leukemia showed sensitivity to VEN (EC_50_ 98.78 nM), while the three *MLL*/*ENL* rearranged leukemias displayed heterogeneous responses with EC_50_ values of 19, 49, and 5499 nM. Similarly, in *ETV6*/*RUNX1* positive ALL (*N* = 6) heterogeneous VEN activities were observed with EC_50_s ranging from 1.87 to 4359 nM. No *TCF3*/*HLF*, *IGH@*/*CRLF2* nor *BCR*/*ABL* positive cases were included and only single cases were positive for *P2RY8*/*CRLF2* or *TCF3*/*PBX1* fusions (Supplementary Fig. 2B). Furthermore, we analyzed copy number alterations recurrently described in BCP-ALL. Most samples were characterized by the presence of at least one copy number alteration (Supplementary Fig. 2C). *CDKN2A* and *CDKN2B* were most frequently affected; however, no differences in VEN sensitivity were observed in samples with or without *CDKN2A* or *CDKN2B* deletions (*U*-test, *p* = 0.867 and *p* = 0.456) and no differences were found for the other aberrations tested. Moreover, the presence of nucleotide variants of genes was also not associated with responses to VEN (Supplementary Fig 2D). Most importantly, we also analyzed *BCL2* but detected wild-type sequences in all cases.

In order to get further insight into potential mechanisms of VEN responsiveness, we analyzed expression profiles of VEN sensitive ALL samples (EC_50_ values below 100 nM, *N* = 12) compared to resistant leukemias (EC_50_ values above 1 µM, *N* = 7). The resulting signature of differentially regulated genes (Supplementary Table 3) did not show enrichment of genes coding for proteins involved in cell death and apoptosis. In addition, pathway annotation studies revealed that the VEN sensitivity/resistance profile was not associated with gene sets annotated to cell death regulation or oncogenic pathways (Supplementary Fig. 3).

### VEN sensitivity is associated with expression levels of mitochondrial apoptosis regulators

Next, we analyzed the impact of expression levels of BCL-2, the direct target molecule of VEN, and of potential mediators of response in BCP-ALL. In a mechanistic concept, VEN binds to BCL-2 with high affinity and selectivity, thereby liberating BCL-2-bound, proapoptotic proteins like BIM leading to induction of downstream apoptosis signaling. In line with this concept, we found that VEN sensitivity correlates with high gene-expression levels of the target molecule BCL-2 (Table 1(a), Supplementary Fig. 4) and as a trend on protein level (Table 1(b), significance level corrected for multiple testing). Interestingly, the most sensitive leukemia with the lowest EC_50_ value (PDX1) showed the highest BCL-2 protein expression (Supplementary Fig. 5). On the other hand, other antiapoptotic BCL-2 family members such as MCL-1, BCL-XL, and BCL-W are able to bind and sequester released proapoptotic regulators like BIM thereby counteracting VEN-induced apoptosis. VEN might also interfere with BCL-XL and BCL-W, but with a clearly lower affinity than with BCL-2^12^. However, high transcript or protein expression of MCL-1, BCL-XL, or BCL-W was not clearly associated with VEN insensitivity (Table 1(a), (b), Supplementary Fig. 4). Balancing the expression levels of BCL-2 and the prosurvival molecules MCL-1, BCL-W, or BCL-XL revealed a significant association of VEN sensitivity with high BCL-2/MCL-1 ratios on transcript and protein level (i.e., high BCL-2/low MCL-1 expression; Table 1(a), (b), Supplementary Fig. 4). Thus, these data indicate that high expression of the target molecule BCL-2, in particular along with low expression of the counteracting molecule MCL-1, is related to VEN-mediated apoptosis induction in BCP-ALL cells. Interestingly, expression of the other prosurvival regulators BCL-XL and BCL-W was not found to be clearly associated with VEN sensitivity, neither alone nor in comparison to BCL-2 (Table 1(a), (b), Supplementary Fig. 4). Moreover, BIM levels were also not associated with VEN sensitivity and the ratio of BCL-2 to BIM did not show a superior association to VEN sensitivity than BCL-2 alone (Supplementary Table 4).

**Table 1:** Expression of BCL-2 family members and ex vivo VEN sensitivity

	*R*^2^	*P*
(a) Gene expression levels^a^		
BCL2/MCL1	0.390	0.0005*
BCL2	0.335	0.0016*
BCL2L2	0.327	0.0018*
MCL1	0.235	0.0104
BCL2/BCL2L1	0.119	0.0783
BCL2/BCL2L2	0.004	0.7608
BCL2L1	0.001	0.9063
(b) Proteins levels^b^		
BCL-2/MCL-1	0.542	0.0041*
BCL-2/BCL-XL	0.431	0.0147
BCL-2	0.354	0.0318
BCL-2/BCL-W	0.328	0.0409
MCL-1	0.243	0.0869
BCL-XL	0.132	0.2218
BCL-W	0.001	0.9405

*R*^2^, correlation coefficient. Bonferroni-adjusted significance level: *P* ≤ 0.05/7 = *P* ≤ 0.0071

*Indicates statistical significance

^a^Patient-derived xenograft BCP-ALL (*N* = 27). Linear regression of EC_50_ of venetoclax with the parameter indicated, sorted by significance

^b^Patient-derived xenograft BCP-ALL (*N* = 13). Linear regression of EC_50_ of venetoclax with the parameter indicated, sorted by significance

Taken together, these data show that sensitivity to VEN is not only determined by presence of the target molecule BCL-2 alone but will rather be affected by the interaction of different BCL-2 family members present in the leukemia cell. However, this regulatory interplay will not be sufficiently reflected by transcript or protein expression of single molecules, but would require a comprehensive analysis of the functional network of different signaling molecules.

### Functional dependence on BCL-2 determines VEN activity

As indicated above, VEN activity in BCP-ALL might be influenced by different regulators of mitochondrial apoptosis signaling. Therefore, we applied an integrative analysis of the functional interplay of the different apoptosis-regulating molecules investigating the dependence of apoptosis signaling on different BCL-2 family members at the mitochondrial level.

**Fig. 2:**
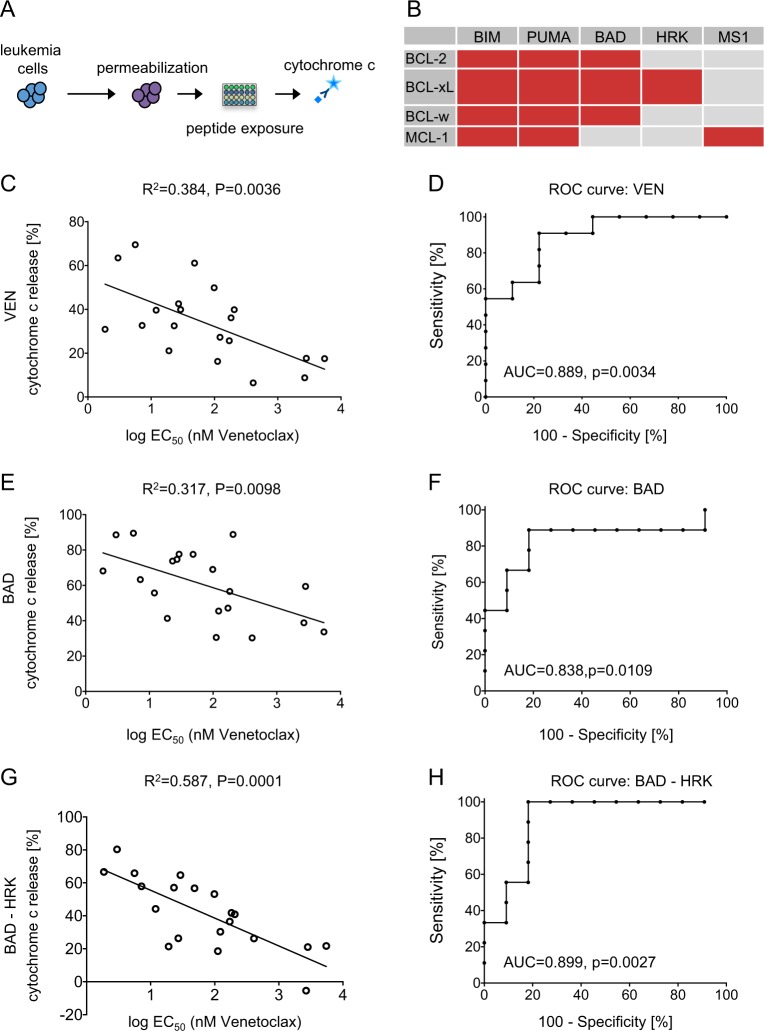
Functional BCL-2 dependence indicates ex vivo VEN activity. Assaying the dependence of mitochondrial apoptosis signaling on different regulating molecules interrogating binding of specific BH3 peptides (BH3 profiling). **a** Experimental procedure, ALL cells are permeabilized, incubated with the respective peptide followed by detection of cytochrome c release. **b** Binding table showing interaction of the BH3-peptides (columns) with the respective apoptosis-regulating molecule (rows). Mitochondrial priming by **c** VEN, **e** BAD, and **g** BAD-HRK is significantly associated with ex vivo venetoclax sensitivity (linear regression; *R*^2^, correlation coefficient; *p*, significance) and **d**, **f**, **h** predictive for ex vivo response of ALL cells to venetoclax (ROC/receiver operating characteristic curve; AUC, area under the curve; *p*, significance)

This method is based on synthetic BH3-only peptides or small molecules, which specifically bind to different BCL-2 family proteins involved in mitochondrial apoptosis regulation (mitochondrial priming)^27–30^ followed by assessment of apoptosis induction assaying mitochondrial cytochrome c release as an early event in apoptosis signaling (Fig. 2a, Supplementary Fig. 6). A panel of five BH3-peptides (BIM, PUMA, BAD, HRK, and MS1) binding to different antiapoptotic molecules, including BCL-2, BCL-xl, BCL-W, and MCL-1, was used (Fig. 2b) and in addition VEN itself. BIM and PUMA bind nonspecifically to antiapoptotic BCL-2 family proteins, BAD binds to BCL-2, BCL-XL and BCL-W, but it does not distinguish between these proteins, and HRK binds to BCL-XL and MS1 to MCL-1^31^. As expected, we found a significant correlation of direct VEN priming with VEN sensitivity obtained upon ex vivo drug exposure and direct VEN priming was predictive for VEN activity ex vivo (Fig. 2c, d). Interestingly, high mitochondrial priming induced by the BAD peptide, which not only binds to BCL-2 but also to BCL-XL and BCL-W, also showed a considerable association with high ex vivo VEN sensitivity, although not gaining statistical significance (Table 2 and Fig. 2e). To correct for the additional binding of the BAD peptide to BCL-XL (Fig. 2b), subtraction of the response induced by HRK from the BAD-response (BAD-HRK) had been described, analyzing the role of BCL-2 and its cellular dependency more precisely^17,18^. Interestingly, BAD-HRK priming showed an association to transcript and protein levels of BCL-2 (Supplementary Table 5) and a strong association to VEN sensitivity (Table 2 and Fig. 2g). Moreover, ex vivo VEN activity in ALL was more precisely predicted by BAD-HRK priming than by priming of BAD alone (Fig. 2f, h). Thus, a strong functional dependence of the leukemia cell on BCL-2 (BAD-HRK priming) is highly indicative for ex vivo anti-ALL activity of VEN. However, binding of antiapoptotic BCL-2 family molecules by BIM and PUMA peptides and, importantly specific interference with MCL-1 and BCL-XL by MS1 or HRK peptide did not show any clear association with VEN sensitivity (Table 2 and Supplementary Fig. 7).

**Table 2:** Association of mitochondrial profiling with ex vivo VEN sensitivity

Parameter	*R*^2^	*P*
BAD-HRK	0.587	0.0001*
VEN	0.384	0.0036*
BAD	0.317	0.0098
PUMA	0.212	0.0413
MS1	0.203	0.0465
HRK	0.140	0.1039
BIM	0.051	0.3404

Patient-derived xenograft BCP-ALL (*N* = 20). Linear regression of EC_50_ of venetoclax with the parameter indicated, sorted by significance. *R*^2^, correlation coefficient. Bonferroni-adjusted significance level: *P* ≤ 0.05/7 = *P* ≤ 0.0071

*Indicates statistical significance

### Preclinical VEN activity is indicated by functional BCL-2 dependence

**Fig. 3:**
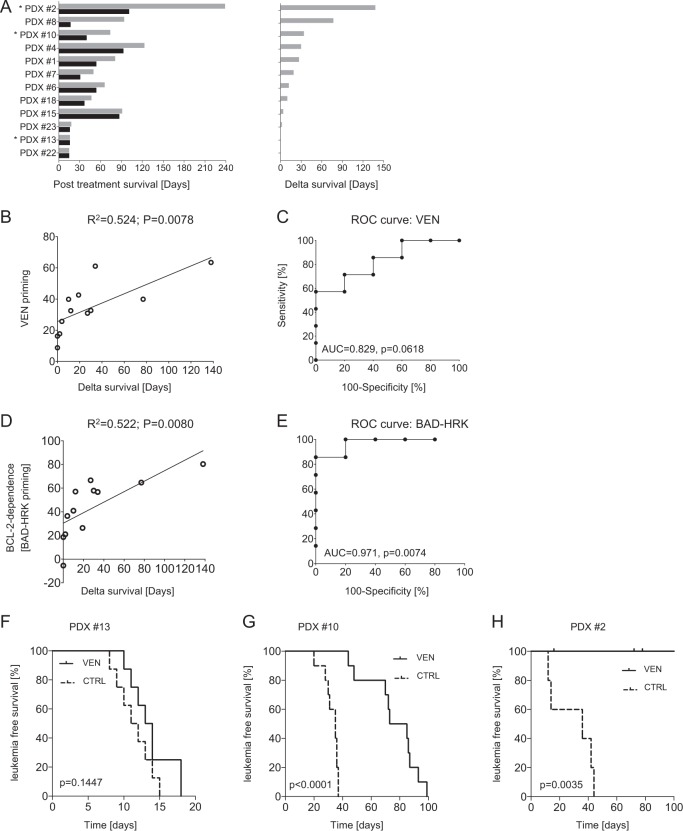
Functional BCL-2 dependence indicates preclinical antileukemia activity in vivo. Individual patient-derived BCP-ALL xenograft samples (*N* = 12) were transplanted onto pairs of recipient mice and treated with either VEN or vehicle for 10 days. After treatment, mice were tightly monitored for onset of leukemia-related morbidity. **a** Survival times of leukemia bearing mice treated with VEN (gray bars) or vehicle (black bars) (left diagram) and corresponding VEN-induced survival differences (‘delta survival’, right diagram). **b** Association of direct VEN priming with preclinical VEN sensitivities of BCP-ALL in vivo (linear regression; *R*^2^, correlation coefficient; *p*, significance) and **c** predictive value of direct VEN priming for post-VEN survival of treated mice (ROC/receiver operating characteristic curve; AUC, area under the curve; *p*, significance). **d** Strong association of functional BCL-2 dependence (mitochondrial BAD-HRK priming) with in vivo VEN responses, and **e** high predictive value of BAD-HRK priming for survival of VEN treated mice. Preclinical in vivo VEN responses analyzed in larger treatment groups confirming **f** low (PDX13; *N* = 8 mice per group), **g** intermediate (PDX10; *N* = 10 mice per group), and **h** strong (PDX2, five mice per group) VEN sensitivity of the respective patient-derived xenograft leukemia observed in the preclinical trial (Kaplan–Meier analysis; *p*, significance by log-rank test; VEN, venetoclax; CTRL control)

Functional BCL-2 dependence (BAD-HRK priming) showed the strongest association with individual VEN sensitivities assessed by ex vivo drug exposure. To address antileukemia activities of VEN in individual leukemia samples in a situation more similar to a potential clinical application, we investigated its antileukemia activities in a preclinical phase-II-like trial on different individual, patient-derived xenograft ALL samples in mice (*N* = 12). Three weeks after transplantation onto recipient mice, ALL-bearing animals were treated with VEN for 10 days and times to reoccurrence of full-blown, clinically apparent leukemia after treatment with VEN or vehicle were compared for each leukemia. We observed distinct in vivo antileukemia activities of VEN indicated by differences of survival times (‘delta survival’) ranging from minimal effects to prolonged survival without manifestation of ALL for more than 140 days (Fig. 3a). This variation of in vivo responses is similar to the heterogeneity of VEN sensitivities observed ex vivo, and EC_50_ values analyzed ex vivo showed a moderate association with in vivo survival times (Table 3, Supplementary Fig. 8).

**Table 3:** Functional dependence on BCL-2 assessed by mitochondrial priming is associated with in vivo VEN activity

*Parameter*	*R*^2^	*P*
VEN	0.524	0.0078*
BAD-HRK	0.522	0.0080*
EC_50_	0.351	0.0423
*BCL2*/*MCL1* (transcript)	0.337	0.0479
BCL-2/MCL-1 (protein)	0.311	0.0597
*BCL2 (transcript)*	0.180	0.1698

In vivo venetoclax treated patient-derived xenograft BCP-ALL (*N* = 12). Linear regression of in vivo response (‘delta survival’) of venetoclax treated mice with the parameter indicated, sorted by significance. *R*^2^, correlation coefficient. Bonferroni-adjusted significance level: *P* ≤ 0.05/6 = *P* ≤ 0.0083

*Indicates statistical significance

We then analyzed whether the molecular markers associated with ex vivo VEN response would indicate in vivo antileukemia activity. No association of preclinical VEN activity with BCL-2 expression was found, neither alone nor relative to *MCL-1* transcript levels (Table 3, Supplementary Fig. 8). However, direct VEN priming was associated with in vivo antileukemia activity of VEN (Table 3 and Fig. 3b), but not predictive for in vivo VEN activity (Fig. 3c). Interestingly, functional dependence of the leukemia cells on BCL-2 (BAD-HRK priming) was strongly associated with in vivo VEN activity (Table 3 and Fig. 3d), and importantly, showed high sensitivity and specificity in predicting preclinical in vivo antileukemia activity of VEN (Fig. 3e).

To further corroborate our findings of the preclinical trial, we investigated three primograft leukemias with distinct responses to VEN (PDX#13, delta survival 0 days; PDX#10, delta survival 34 days; and PDX#2, delta survival > 138 days) in larger experimental treatment groups of 5–10 recipients. Upon leukemia manifestation (presence of 5% human ALL cells in the recipients peripheral blood), mice were treated with either VEN or vehicle for 10 days followed by assessment of leukemia-free survival until disease manifestation for each recipient. These results obtained from larger groups of biological replicates precisely reflected the drug responses seen in the preclinical trial and, importantly, clearly corresponded to the degree of BCL-2 dependence assessed by mitochondrial priming: (i) PDX13 showed a minor delay of disease manifestation and low BCL-2 dependence (Fig. 3f, mean survival difference 2.3 days, BAD-HRK priming 18.6%), (ii) in PDX10 we observed a significantly delayed onset of overt leukemia upon VEN therapy in line with clear BCL-2 dependence (Fig. 3g, mean survival difference 43.2 days, BAD-HRK priming 56.8%), and (iii) PDX2 showed a prolonged survival with no leukemia manifestation in the VEN group within the observation period (Fig. 3h, more than 70 days superior survival, BAD-HRK priming 80.3%) corresponding to a strong BCL-2 dependence.

**Fig. 4:**
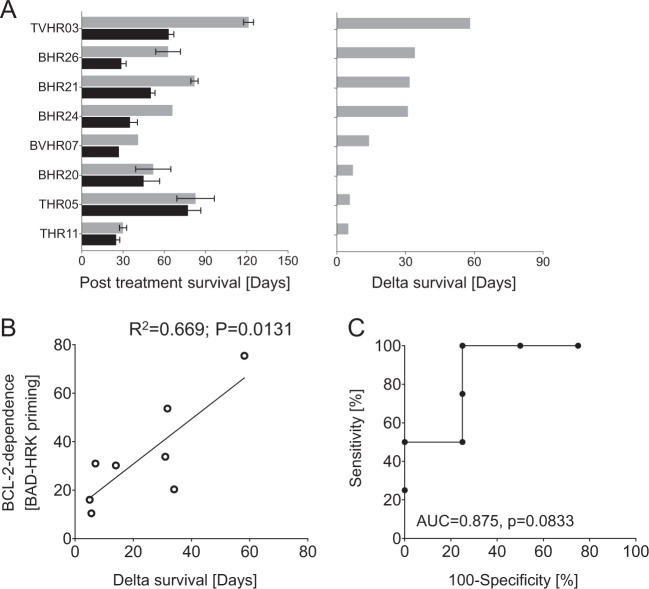
Functional BCL-2 dependence indicates in vivo preclinical antileukemia activity in an independent cohort of patient-derived ALL samples. Individual patient-derived ALL xenograft samples (BCP-ALL *N* = 5, T-ALL *N* = 3) were transplanted onto groups of recipient mice and treated with either VEN or vehicle. After treatment, mice were regularly monitored for the appearance of leukemia cells (≥5% or more of mCD45^−^huCD45^+^huCD19^+^ or huCD7^+^) in the peripheral blood. **a** Survival times of leukemia bearing mice treated with VEN (gray bars) or vehicle (black bars, left diagram) and corresponding VEN-induced survival differences (‘delta survival’, right diagram). **b** Significant association of functional BCL-2 dependence (mitochondrial BAD-HRK priming) with preclinical VEN sensitivities of ALL in vivo (linear regression; *R*^2^, correlation coefficient; *p*, significance) and **c** high predictive value for post-VEN survival (ROC/receiver operating characteristic curve; AUC, area under the curve; *p*, significance)

Moreover, we also analyzed mitochondrial priming in leukemia cells of an independent cohort of patient-derived ALL primografts, in which in vivo antileukemia activity of VEN had been analyzed in a similar preclinical xenograft mouse model^21,32^. This cohort of eight patient-derived ALL samples comprises both, BCP- (*N* = 5) and T- (*N* = 3) ALL samples, and also includes three BCP-ALL cases carrying a *TCF3*/*HLF* gene fusion (Supplementary Table 6), a genetic aberration associated with very poor prognosis. Comparing the survival times of VEN- and vehicle-treated animals, we found different times indicating distinct in vivo VEN sensitivities of the individual leukemias (Fig. 4a). Most importantly, also in this cohort we found that functional BCL-2 dependence of the leukemia cells measured by BAD-HRK priming is significantly associated with preclinical response (Fig. 4b) predicting in vivo antileukemia VEN activity, in both BCP- and T-ALL (Fig. 4c).

Thus, our findings show that interrogation of mitochondria with BH-3 mimetic peptides in ALL yields molecular information on pathways providing criteria for upfront identification of patients responding to BCL-2 directed therapy, being of relevance for patient treatment stratification. Moreover, the advantage of a rapid analysis time and quick availability of the result within few hours strongly emphasizes the use and prospective evaluation of this marker in future clinical application of VEN in ALL.

## Discussion

The orally bioavailable BCL-2 inhibitor VEN has proven effectiveness in chronic lymphocytic leukemia (CLL) and other hematological malignancies^11,14–18^, and has been implemented into regimens treating CLL patients^33^. In ALL, application of VEN has just started in first clinical trials investigating activity in relapsed or refractory disease (ClinicalTrials.gov)^24^; however clinical response data, particularly for de novo ALL, are not yet available. Thus, rapid identification of patients who would benefit from BCL-2-directed treatment is of major importance and markers which best reflect antileukemia activity of VEN in the situation of clinical application are strongly needed.

Investigating a series of 27 patient-derived BCP-ALL samples, we titrated half maximal EC_50_ for each individual leukemia. Variable responses were observed in different patient-derived xenograft BCP-ALL samples with high sensitivity at nanomolar drug concentrations and lower sensitivity or no response, in line with heterogeneous VEN sensitivities previously reported in BCP-ALL^19,21,34^. Given this heterogeneity of responses to VEN among different individual leukemias, we aimed to analyze markers indicating sensitivity to BCL-2 inhibition.

Clinical markers of the different leukemias established for prognosis and therapy stratification were not associated with VEN sensitivity. In particular, recurrent genetic alterations described in BCP-ALL including aberrations in *IKZF1*, *CDKN2A*/*B*, and *PAX5* were not related to sensitivity. Previous reports described *TCF3*/*HLF* rearranged BCP-ALL to be particularly sensitive to VEN^21,32^, a rare BCP-ALL subgroup with dismal outcome^35^. However none of the leukemias investigated in our initial analysis cohort carried this gene fusion. The *MLL*/*AF4* fusion was described to mediate high BCL-2 expression leading to VEN sensitivity^34^, and response was reported in four out of four samples tested in an ex vivo drug screen and in three out of six *MLL*/*AF4* positive leukemias upon in vivo treatment^21,36^. In our study, one *MLL*/*AF4* positive case was included, which was responsive to VEN. On the other hand, two out of three *MLL*/*ENL* rearranged cases were sensitive while one was the most insensitive case in our cohort. Thus, in addition to potential sensitivities in particular subgroups, we and others showed various VEN activities across different BCP-ALL subgroups including high sensitivities in many cases, which are not characterized by established diagnostic markers^21,34,36^, further underlining the need for independent determinants indicating VEN response.

VEN binds directly to BCL-2, leading to release of proapoptotic molecules and apoptosis induction^12^. In line, we found higher expression levels of the target molecule BCL-2 in responsive leukemias, consistent with previously reported results in BCP-ALL, T-ALL, and AML cell lines^34,37,38^. Other BCL-2 family members like MCL-1 and BCL-XL sequester proapoptotic regulators released from BCL-2, thereby counteracting apoptosis induction^13^. In line with this mechanism, we found low MCL-1 together with high BCL-2 expression to be associated with VEN sensitivity. Accordingly, high BCL-2 relative to low MCL-1 expression was reported to indicate BCL-2 inhibitor sensitivity in multiple myeloma, mantle cell lymphoma and Philadelphia-chromosome positive ALL^39–41^. However, other studies did not observe associations of BCL-2 or MCL-1 expression and drug sensitivity in ALL^21,34^. BCL-XL, another antiapoptotic molecule, was described to have regulatory impact counteracting sensitivity in a study investigating VEN in ALL xenografts in vivo^36^. In contrast to this, other studies did not observe an association of BCL-XL and VEN activity, as we did also not find a clear connection of drug sensitivity with BCL-XL in our cohort^21,34^. Analyzing expression of proapoptotic BIM, we did not find an association with VEN sensitivity in our ALL model, suggesting that BIM levels do not sufficiently explain VEN activity and emphasizing that a more complex interplay of apoptosis regulators determines drug sensitivity, as recently proposed for other BH3-mimetics^42^. Thus, although indicative for single molecules, these different findings and variations of expression levels observed in distinct studies clearly underline the limitations of quantifying levels of single regulators or ratios as reliable indicators for drug response.

Key apoptosis signaling pathways converge at the mitochondrial level and are controlled by different proapoptotic and antiapoptotic molecules constituting a functional regulatory network. Consequently, the activity of therapeutic agents targeting specific mitochondrial regulators, like VEN targets BCL-2, is not only determined by direct interference, but rather a result of the interplay of involved molecules. In an integrative analysis, we therefore interrogated mitochondrial apoptosis regulation using a panel of synthetic proapoptotic BH3-peptides, which interact with the respective antiapoptotic molecules and delineate their specific roles and the cell’s addiction to particular regulators of mitochondrial apoptosis signaling^43^. Analyzing the early apoptosis signaling event mitochondrial cytochrome c release as a readout, this assay provides results within few hours and does not require culture of primary patient cells, which might bias the analysis by high cell death rates upon prolonged culture of primary ALL cells. We applied this functional approach and observed that leukemia cells with strong functional dependence on BCL-2 (BAD-HRK priming) showed high VEN sensitivity upon ex vivo exposure (low EC_50_ values), in line with previous reports on BCL-2 dependence and VEN sensitivity measured in isolated BCP-ALL and T-ALL cells^17–19,34^. However, assessing drug sensitivities in isolated primary ALL cells upon prolonged ex vivo culture is limited by high cell death rates due to lacking or reduced cellular contact and survival signals by a “death by default” mechanism^44,45^. In line, clinical patient outcome upon specific treatment is more accurately reflected by response evaluation of patient-derived ALL modeled in vivo in a phase-II-like preclinical trial^46^. Preclinical in vivo response was found to be associated with direct VEN priming and, as previously reported, with ex vivo measured VEN response^21,36^. However, comparing the associations and predictive values of markers identified for ex vivo sensitivity, we found that a clear dependency of mitochondrial signaling on BCL-2 in the leukemia cell is strongly associated with high response rates in vivo and, most importantly, most accurately predicts leukemia-free survival of BCP-ALL. Importantly, this finding of a highly indicative value of functional BCL-2 dependence for in vivo VEN sensitivity was confirmed in an independent set of preclinically treated primograft samples and extended to T-ALL and *TCF3/HLF* rearranged ALL, a rare subgroup associated with early relapse and dismal outcome^35^, for which in vitro and preclinical in vivo response to VEN had recently been reported^32^.

Taken together, our findings underline the importance to further investigate markers, which identify patients who would benefit from BCL-2-directed therapy. In our study, we show that assaying the leukemia cell’s addiction to BCL-2-mediated mitochondrial apoptosis signaling is a rapid procedure providing biological information available in a short time accurately predicting VEN sensitivity, which can be used for upfront identification of responsive patients and clinical guidance in future trials evaluating VEN efficacy in BCP-ALL.

## Materials and methods

### ALL cell lines

Cell lines were obtained from DSMZ, Braunschweig, Germany, UoCB6 cells were kindly provided by Dr J. Rowley, Chicago, USA. Initially, cells were amplified, authenticated by short tandem repeat profiling and tested for Mycoplasma negativity by DAPI staining (5 min DAPI-methanol 1:5000 in sterile PBS at room temperature, followed by washing with sterile PBS and microscopy) and stocks were frozen. For experiments, cells were thawed and used for analyses within 40 passages. Cells were cultured in RPMI 1640 medium supplemented with 10–20% fetal bovine serum and L-Glutamine (Gibco Life Technologies, Germany) at 37 °C in a humidified atmosphere with 5% carbon dioxide.

## ALL xenograft samples

Leukemia samples were obtained from pediatric patients diagnosed with precursor B-ALL after informed consent of patients and/ or their legal guardians in accordance with the institution’s ethical review board. All patients have been diagnosed and treated according to the AIEOP-BFM protocols^1^. Primograft leukemias were established by transplanting ALL cells onto female NOD/SCID mice (NOD.CB17-Prkdcscid, Charles River, Germany) as described earlier^47,48^. Immunophenotyping was carried out following standard procedures analyzing cells on a LSR II flow cytometer (BD Bioscience, Germany). All animal experiments were approved by the appropriate authority (Regierungspräsidium Tübingen, Tierversuch Nr. 1260).

### Cell viability assays

Cell viability assays were performed upon culturing of cells in RPMI 1640 supplemented with 20% FCS and 1% l-glutamine. Cells were exposed to 11 different concentrations of VEN (0.1 nM, 1 nM, 10 nM, 50 nM, 100 nM, 250 nM, 500 nM, 1 µM, 3 µM, 5 µM, and 10 µM) for 72 h (BCP-ALL cell lines) or 24 h (BCP-ALL PDX cells). VEN was purchased from Chemietek (USA). Cell viability and cell death were assessed on a BD FACSCalibur using forward/side scatter criteria. Half maximal EC_50_ of VEN were analyzed for each sample.

### In vivo treatment

Upon transplantation of ALL cells, engraftment of human blasts was monitored in peripheral blood by flow cytometry surface staining for huCD19 and huCD45^49,50^. Mice were treated with vehicle (60% Phosal 50 PG, 30% polyethylene glycol and 10% ethanol) or VEN 100 mg/kg/day orally for 10 days. Treatment was initiated on day 21 post transplantation (Fig. 3a) or upon engraftment of more than 5% blasts in the peripheral blood (Fig. 3f–h). Posttreatment survival times were defined as manifestation of clinically overt leukemia in recipient animals upon initiation of treatment. Manifestation of leukemia was confirmed by flow cytometry staining of bone marrow and spleen cells as described above showing high percentages of human ALL in the respective compartments. For the independent cohort (Fig. 4) treatment was carried out as previously described^21,32^.

### Western blot analysis

Western blotting was performed as previously described^48^. BCL-2 Mouse monoclonal antibody (BD Biosciences, Germany), Mcl-1 Rabbit polyclonal antibody (Stressgen, Canada), Bcl-w Rabbit monoclonal antibody (Cell Signaling Technology, Germany), Bcl-xl Rabbit monoclonal antibody (Cell Signaling Technology, Germany), ß-Actin Mouse mononoclonal antibody, (Sigma-Aldrich, Germany) and mouse anti-alpha-Tubulin monoclonal antibody (Calbiochem, USA) were used as primary antibodies, followed by goat anti-mouse IgG-HRP or goat anti-rabbit IgG-HRP (Santa Cruz Biotechnology, USA) secondary antibodies.

### Genetic alterations

Deletions and amplifications were assessed by Multiplex Ligation-dependent Probe Amplification (MLPA; SALSA MLPA P335-B2 ALL-IKZF1 probemix, MRC-Holland, The Netherlands) according to the manufacturer’s instructions. P2RY8-CRLF2 (primer: 5′-GGACAGATGGAACTGGAAGG-3′ and 5′-GTCCCATTCCTGATGGAGAA-3′), IGH@-CRLF2 (primer: 5′- AATACTTCCAGCACT-3′ and 5′-GTCCCATTCCTGATGGAGAA-3′), TCF3/PBX1 (primer: 5′-CACCAGCCTCATGCACAAC-3′ and 5′-TCGCAGGAGATTCATCACG-3′) and TCF3/HLF (primer: 5′-TCCAGCCCTTCTACCCCCGTGG-3′ and 5′-GCATTTGCCCAGCTCCTTCCTCAA-3′) were analyzed by RT-PCR as described earlier^51^. DNA sequencing was performed using the TruSeq custom amplicon kit (Illumina, San Diego, USA) according to the manufacturer’s instructions. Reads were aligned to the hg19 human reference genome. Variant calling files were generated and custom R scripts were used for further downstream analyses. Only variants with a variant allele frequency cutoff of 0.2 were considered. For detection of *TP53* mutations, exons 4 to 10 (coding region of P53) were analyzed by denaturing high-performance liquid chromatography on a WAVE 3500HT System (ADS Biotec, Glasgow, UK)^52^.

### Gene-expression analysis

Gene expression was analyzed using Affymetrix U133 Plus 2.0 arrays as described before^53^. Gene-expression data were deposited in the Gene Expression Omnibus database of the National Center for Biotechnology Information (https://www.ncbi.nlm.nih.gov/geo/query/acc.cgi?acc=GSE123883, GEO series accession number GSE123883). To determine the expression of single genes, the geometric mean of all probe sets mapping a gene was calculated and log2 median-centered expression values were analyzed. The following probe sets were used to analyze transcript levels of apoptosis regulators (*BCL2:* 207004_at, 207005_s_at, 203684_s_at, and 203685_at), (*MCL1:* 200796_s_at, 200797_s_at, 200798_x_at, 214056_at, 214057_at, and 227175_at), (*BCL2L1:* 206665_s_at, 231228_at, 212312_at, and 215037_s_at), (*BCL2L2:* 1555140_a_at, and 209311_at), and (*BCL2L11:* 1553096_s_at, 222343_at, 1558143_a_at, 225606_at, 1553088_a_at, 208536_s_at, and 1555372_at).

### BH3 profiling and intracellular flow cytometry

Intracellular BH3 profiling was performed as described earlier by measuring mitochondrial cytochrome c release by flow cytometry^43^. Cryopreserved pediatric ALL PDX samples were thawed, permeabilized with digitonin and exposed to proapoptotic BH3 peptides or venetoclax before fixation in formaldehyde. Cells were stained with anti-cytochrome c antibody Alexa Fluor 488 (#612308, Biolegend). The peptide-induced cytochrome c release was quantified as median fluorescence intensity (MFI) normalized to the MFIs of alamethicin (positive control of cytochrome c release; #BML-A150-0005, Enzo) and DMSO (negative control). For intracellular protein staining, cells were stained with anti-BCL-2 mAb Alexa Fluor 488 (#59422, Cell Signaling), anti-BIM mAb Alexa Fluor 488 (#94805, Cell Signaling), mouse mAb IgG1 Isotype Control Alexa Fluor 488 (#4878, Cell Signaling) and rabbit IgG Isotype Control Alexa Fluor 488 (#4340, Cell Signaling). MFIs were quantified and normalized to the MFIs of the respective isotype controls.

### Statistical analysis

Data were analyzed using GraphPad Prism software. Mann–Whitney test was used to compare values of two groups, linear regression to test an association of two variables to each other, and survival analyses were performed using the Kaplan–Meier method and the log-rank test.

## Supplementary information

Supplementary data
