# Supplementary material for: Prediction of venetoclax activity in precursor B-ALL by functional assessment of apoptosis signaling
Source: Cell Death Dis. 2019 Jul 29;10(8):571. doi: 10.1038/s41419-019-1801-0 (PMC6662703; doi:10.1038/s41419-019-1801-0)
Supplement: Supplementary file 1 — Supplementary data [file 41419_2019_1801_MOESM1_ESM.docx]

Seyfried *et al*.

Prediction of Venetoclax Activity in Precursor B-ALL by Functional Assessment of Apoptosis Signaling

**Supplementary Information:**

**Supplementary Tables**

**Supplementary Table 1: Characteristics of BCP-ALL cell lines**

| **Cell line** | **Genetic alterations** | **VEN EC_50_ [nM]** |
| --- | --- | --- |
| RS4;11 | MLL-AF4[^1^](#_ENREF_1)^,^[^2^](#_ENREF_2) | 24 |
| KOPN-8 | MLL-ENL[^1^](#_ENREF_1) | 148 |
| UoCB6 | ETV6-RUNX1[^3^](#_ENREF_3) | 376 |
| REH | ETV6-RUNX1[^1^](#_ENREF_1) | 438 |
| RCH-ACV | TCF3-PBX1[^1^](#_ENREF_1)^,^[^4^](#_ENREF_4) | 6839 |
| Nalm-6 | ETV6-PDGFRB[^1^](#_ENREF_1)^,^[^5^](#_ENREF_5) | 11262 |

**Supplementary Table 2: EC_50_-values of BCP-ALL xenograft samples**

| PDX | VEN EC_50_ [nM] |
| --- | --- |
| PDX1 | 2 |
| PDX2 | 3 |
| PDX3 | 6 |
| PDX4 | 7 |
| PDX5 | 12 |
| PDX6 | 19 |
| PDX7 | 23 |
| PDX8 | 27 |
| PDX9 | 29 |
| PDX10 | 48 |
| PDX11 | 61 |
| PDX12 | 99 |
| PDX13 | 112 |
| PDX14 | 122 |
| PDX15 | 171 |
| PDX16 | 183 |
| PDX17 | 190 |
| PDX18 | 206 |
| PDX19 | 377 |
| PDX20 | 410 |
| PDX21 | 2224 |
| PDX22 | 2665 |
| PDX23 | 2831 |
| PDX24 | 3122 |
| PDX25 | 3729 |
| PDX26 | 4359 |
| PDX27 | 5499 |

**Supplementary Table 3: Differentially expressed genes comparing venetoclax-sensitive and -resistant xenograft samples**

Gene expression was analyzed comparing venetoclax-sensitive (EC_50_ < 100 nM, n=12) and -resistant (>1 µM, n=7) samples. 39 probe sets were found to be up-regulated in venetoclax-resistant and 33 probe sets were up-regulated in venetoclax-sensitive samples. Shrinkage t-test, fold change (FC) > 1.6 (given as logarithm, base 2), FDR < 0.1.

**Up-regulated in Venetoclax-resistant Up-regulated in Venetoclax-senstitive**

| probe set | hugo | fc | qval | probe set | hugo | fc | qval |
| --- | --- | --- | --- | --- | --- | --- | --- |
| 227364_at | 227364_at | -3.526 | 0.080 | 205655_at | MDM4 | 2.954 | 0.098 |
| 227621_at | WTAP | -2.629 | 0.087 | 239516_at | 239516_at | 2.913 | 0.094 |
| 235589_s_at | 235589_s_at | -2.548 | 0.092 | 211022_s_at | ATRX | 2.731 | 0.091 |
| 229026_at | CDC42SE2 | -2.534 | 0.099 | 213472_at | HNRNPH1 | 2.710 | 0.099 |
| 229905_at | RAP1GDS1 | -2.371 | 0.092 | 214657_s_at | 214657_s_at | 2.644 | 0.090 |
| 213979_s_at | 213979_s_at | -2.228 | 0.097 | 233393_at | 233393_at | 2.136 | 0.079 |
| 229067_at | 229067_at | -2.110 | 0.064 | 207564_x_at | OGT | 2.131 | 0.050 |
| 228638_at | FAM76A | -2.104 | 0.085 | 244230_at | 244230_at | 2.069 | 0.050 |
| 228443_s_at | KMT5A | -2.067 | 0.030 | 210365_at | 210365_at | 2.002 | 0.088 |
| 228196_s_at | LARP4B | -2.049 | 0.092 | 244030_at | STYX | 1.933 | 0.042 |
| 231169_at | 231169_at | -2.045 | 0.089 | 238736_at | REV3L | 1.923 | 0.093 |
| 214041_x_at | RPL37A | -1.821 | 0.072 | 202731_at | 202731_at | 1.797 | 0.099 |
| 228204_at | PSMB4 | -1.801 | 0.050 | 220553_s_at | PRPF39 | 1.795 | 0.097 |
| 1563431_x_at | CALM3 | -1.738 | 0.088 | 202730_s_at | 202730_s_at | 1.668 | 0.096 |
| 213789_at | EBP | -1.728 | 0.030 | 222031_at | LOC389906 | 1.622 | 0.090 |
| 231101_at | 231101_at | -1.689 | 0.100 | 215985_at | 215985_at | 1.527 | 0.089 |
| 228251_at | UBXN6 | -1.656 | 0.077 | 231955_s_at | HIBADH | 1.481 | 0.091 |
| 213619_at | HNRNPH1 | -1.613 | 0.087 | 1555981_at | ASB16-AS1 | 1.474 | 0.084 |
| 228588_s_at | UBE2B | -1.602 | 0.048 | 243047_at | FAM193B | 1.408 | 0.093 |
| 235819_at | BTF3L4 | -1.591 | 0.091 | 213584_s_at | CREBZF | 1.392 | 0.095 |
| 213536_s_at | UBE2I | -1.543 | 0.092 | 243826_at | 243826_at | 1.379 | 0.086 |
| 241941_at | 241941_at | -1.504 | 0.082 | 224522_s_at | DCAKD | 1.355 | 0.087 |
| 213928_s_at | AGFG1 | -1.299 | 0.090 | 220368_s_at | PPP4R3A | 1.246 | 0.088 |
| 207688_s_at | INHBC | -1.266 | 0.096 | 212515_s_at | DDX3X | 1.236 | 0.093 |
| 216210_x_at | TRIOBP | -1.261 | 0.098 | 201132_at | 201132_at | 1.186 | 0.084 |
| 213956_at | CEP350 | -1.234 | 0.074 | 208860_s_at | ATRX | 1.154 | 0.081 |
| 210733_at | TRAM1 | -1.228 | 0.030 | 208698_s_at | NONO | 1.152 | 0.062 |
| 231004_s_at | 231004_s_at | -1.157 | 0.030 | 236910_at | MRPL39 | 1.051 | 0.096 |
| 1555272_at | 1555272_at | -1.032 | 0.089 | 210470_x_at | NONO | 1.009 | 0.080 |
| 203945_at | ARG2 | -0.999 | 0.097 | 223160_s_at | PPP4R3A | 1.002 | 0.030 |
| 222437_s_at | 222437_s_at | -0.969 | 0.072 | 243631_at | 243631_at | 0.883 | 0.098 |
| 219198_at | GTF3C4 | -0.954 | 0.093 | 200692_s_at | HSPA9 | 0.784 | 0.079 |
| 211519_s_at | KIF2C | -0.791 | 0.092 | 1558546_at | DNASE1 | 0.766 | 0.089 |
| 217947_at | CMTM6 | -0.791 | 0.087 |  |  |  |  |
| 221864_at | ORAI3 | -0.738 | 0.082 |  |  |  |  |
| 239294_at | PIK3CG | -0.725 | 0.090 |  |  |  |  |
| 224241_s_at | 224241_s_at | -0.716 | 0.086 |  |  |  |  |
| 1553858_at | ZBTB3 | -0.693 | 0.091 |  |  |  |  |
| 218038_at | DMAC2 | -0.685 | 0.099 |  |  |  |  |

**Supplementary Table 4: Association of apoptosis molecules with VEN activity**

(**A**) Association (linear regression) of indicated apoptosis regulating molecules with EC_50_s of VEN. R^2^, correlation coefficient. P, significance.

|  | *Protein level* | N | R^2^ | P |
| --- | --- | --- | --- | --- |
|  | BIM | 27 | 0.081 | 0.1500 |
|  | BCL-2 | 27 | 0.309 | 0.0026 |
|  | BCL2/BIM | 27 | 0.287 | 0.0040 |

|  | *Transcript level* | N | R^2^ | P |
| --- | --- | --- | --- | --- |
|  | *BCL2L11 (BIM)* | 27 | 0.012 | 0.5796 |
|  | *BCL2* | 27 | 0.335 | 0.0016 |
|  | *BCL2/BCL2L11* | 27 | 0.204 | 0.0182 |

(**B**) Association of indicated apoptosis regulators with delta survival of mice treated with VEN (see also Figure 3). R^2^, correlation coefficient. P, significance.

|  | *Transcript level* | N | R^2^ | P |
| --- | --- | --- | --- | --- |
|  | *BCL2L11 (BIM)* | 12 | 0.125 | 0.2607 |
|  | *BCL2* | 12 | 0.180 | 0.1698 |
|  | *BCL2/BCL2L11* | 12 | 0.254 | 0.0947 |

|  | *Protein level* | N | R^2^ | P |
| --- | --- | --- | --- | --- |
|  | BIM | 12 | 0.117 | 0.2776 |
|  | BCL-2 | 12 | 0.288 | 0.0718 |
|  | BCL2/BIM | 12 | 0.342 | 0.0475 |

**Supplementary Table 5: Expression levels of BCL-2 family members and corresponding BH3 profiling parameters**

(**A**) Association (linear regression) of transcript levels of indicated apoptosis regulating molecules with BH3 profiling parameters. N = 20, R^2^, correlation coefficient. P, significance.

|  | Parameters correlated | |  |  |
| --- | --- | --- | --- | --- |
|  | **functional priming** | **transcript expression** | **R^2^** | **P** |
|  | BAD-HRK | *BCL2* | 0.536 | 0.0002 |
|  | HRK | *BCL2L1* | 0.101 | 0.1718 |
|  | MS1 | *MCL1* | 0.002 | 0.8500 |

|  | Parameters correlated | |  |  |
| --- | --- | --- | --- | --- |
|  | **functional priming** | **protein expression** | **R^2^** | **P** |
|  | BAD-HRK | BCL-2 | 0.665 | 0.0007 |
|  | HRK | BCL-XL | 0.015 | 0.6948 |
|  | MS1 | MCL-1 | 0.004 | 0.8387 |

(**B**) Association (linear regression) of protein levels of indicated apoptosis regulating molecules with BH3 profiling parameters. N = 13, R^2^, correlation coefficient. P, significance.

**Supplementary Table 6: Characteristics of ALL xenograft samples (independent cohort)**

| **PDX** | **Genetic alterations** | **Immunophenotype** |
| --- | --- | --- |
| THR11 | CDKN2A/B del, NOTCH1mut | Cortical T |
| THR05 | CDKN2A/B del, NOTCH1mut | Cortical T |
| BHR20 | TCF3-HLF | Common BCP |
| BVHR07 | MLL-AF4 | Pro BCP |
| BHR24 | TCF3-HLF | Common BCP |
| BHR21 | TCF3-HLF | Common BCP |
| BHR26 | MLL-AF4 | Pro BCP |
| TVHR03 | CDKN2A/B del | Cortical T |

**Supplementary Figures:**

**Supplementary Figure 1: Venetoclax sensitivity remains stable upon serial transplantation in patient-derived xenografts (PDXs)**

(**A**) Venetoclax sensitivity of PDX10 was assessed in three different passages (p1, p2 and p3) upon serial transplantation. Cell death was analyzed by forward/side scatter criteria, exposure for 24 hours to increasing concentrations: 0.1 nM, 1 nM, 10 nM, 50 nM, 100 nM, 250 nM, 500 nM, 1 µM, 3 µM, 5 µM and 10 µM). EC_50_-values were calculated (PDX10 p1: EC_50_ = 49.81 nM, p2: EC_50_ = 35.44 nM and p3: EC_50_ = 38.06 nM). (**B**) Venetoclax sensitivity of PDX13 was assessed in three different xenograft passages (p4, p5 and p6) (concentrations of venetoclax and assessment of cell death rates as in A, drug exposure time 24 hours). EC_50_-values were determined (PDX13 p4: EC_50_ = 108.4 nM, p5: EC_50_ = 96.72 nM and p6: EC_50_ = 137.6 nM.

A B

PDX10 p1

PDX10 p2

PDX10 p3

PDX13 p4

PDX13 p5

PDX13 p6

relative cell death [%]

Venetoclax [24 h, nM]

relative cell death [%]

Venetoclax [24 h, nM]

**Supplementary Figure 2: Venetoclax activity is not associated with leukemia characteristics**

(**A**) Patient and leukemia characteristics of N=27 patient-derived xenograft BCP-ALL samples. (**B**) Recurrent gene fusions, (**C**) copy number alterations, and (**D**) nucleotide variants are not associated with venetoclax sensitivity in BCP-ALL (increasing EC_50_-values from left to right, top panel).

**Supplementary Figure 3: Venetoclax-sensitivity /-resistance is not associated with oncogenic pathways**

Gene expression was analyzed using Affymetrix U133 Plus 2.0 arrays. Gene set enrichment analysis (GSEA, http://software.broadinstitute.org/gsea/msigdb/index.jsp) was performed comparing venetoclax-sensitive (green, EC_50_ < 100 nM, n=12) and -resistant (red, >1 µM, n=7) samples. GSEA using 186 KEGG gene sets did not identify any significant enrichment in both groups. The heat map shows the expression of oncogenes and tumor suppressor genes annotated to KEGG gene sets.

Apoptosis

Cell Cycle

Jak-STAT Signaling

MAPK Signaling

B-Cell Receptor

Signaling

**Supplementary Figure 4: Expression of BCL-2 family members and *ex vivo* venetoclax sensitivity**

(**A**) Association (linear regression) of EC_50_ of venetoclax with transcript expression of the indicated apoptosis regulating molecules. N=27 patient-derived xenograft BCP-ALL. R^2^, correlation coefficient. P, significance, Bonferroni-adjusted significance level: P ≤ 0.05/7 = P ≤ 0.0071. See also Table 1A.

*BCL2 / MCL1*

[log2 median-centered

transcript ratio]

*BCL2L2*

[log2 median-centered

transcript]

*BCL2 / BCL2L1*

[log2 median-centered

transcript ratio]

log EC_50_ (nM Venetoclax)

**R^2^=0.390, P=0.0005**

R^2^=0.327, P=0.0018

R^2^=0.119, P=0.0783

*BCL2 / BCL2L2*

[log2 median-centered

transcript ratio]

*MCL1*

[log2 median-centered

transcript]

*BCL2*

[log2 median-centered

transcript]

**R^2^=0.335, P=0.0016**

R^2^=0.235, P=0.0104

R^2^=0.004, P=0.7608

*BCL2L1*

[log2 median-centered

transcript ratio]

R^2^=0.001, P=0..9063

**Supplementary Figure 4 contd.:**

(**B**) Association (linear regression) of EC_50_ of venetoclax with protein expression of the indicated apoptosis regulating molecules. N=13 patient-derived xenograft BCP-ALL. R^2^, correlation coefficient. P, significance, Bonferroni-adjusted significance level: P ≤ 0.05/7 = P ≤ 0.0071. See also Table 1B.

BCL-2 / MCL-1

[log2 median-centered

protein ratio]

BCL-2

[log2 median-centered

protein expression]

MCL-1

[log2 median-centered

protein expression]

log EC_50_ (nM Venetoclax)

**R^2^=0.542, P=0.0041**

R^2^=0.354, P=0.0318

R^2^=0.243, P=0.0869

BCL-XL

[log2 median-centered

protein expression]

BCL-2 / BCL-W

log2 median-centered

protein ratio]

R^2^=0.431, P=0.0147

R^2^=0.328, P=0.0409

R^2^=0.132, P=0.2218

BCL-W

[log2 median-centered

protein expression]

R^2^=0.001, P=0.9405

BCL-2 / BCL-XL

[log2 median-centered

protein ratio]

**Supplementary Figure 5: Protein levels of BCL-2 family members**

Western blot analysis of basal protein levels of members of the BCL-2 family used for densitometric quantification (Table 1b).

Cell lines Patient-derived xenografts (PDX)


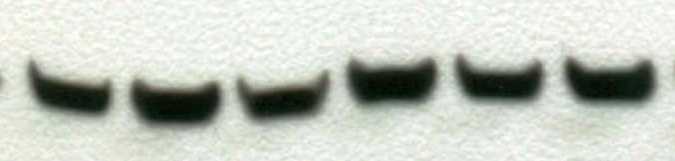

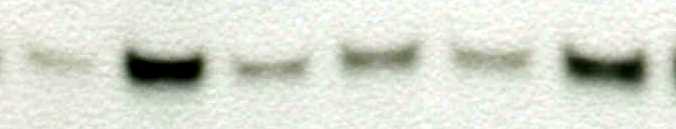

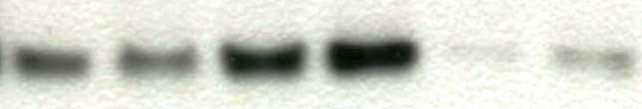


KOPN-8

RS4;11

REH

UoCB-6

NALM-6

BCL-2

MCL-1

α-TUBULIN

RCH-ACV


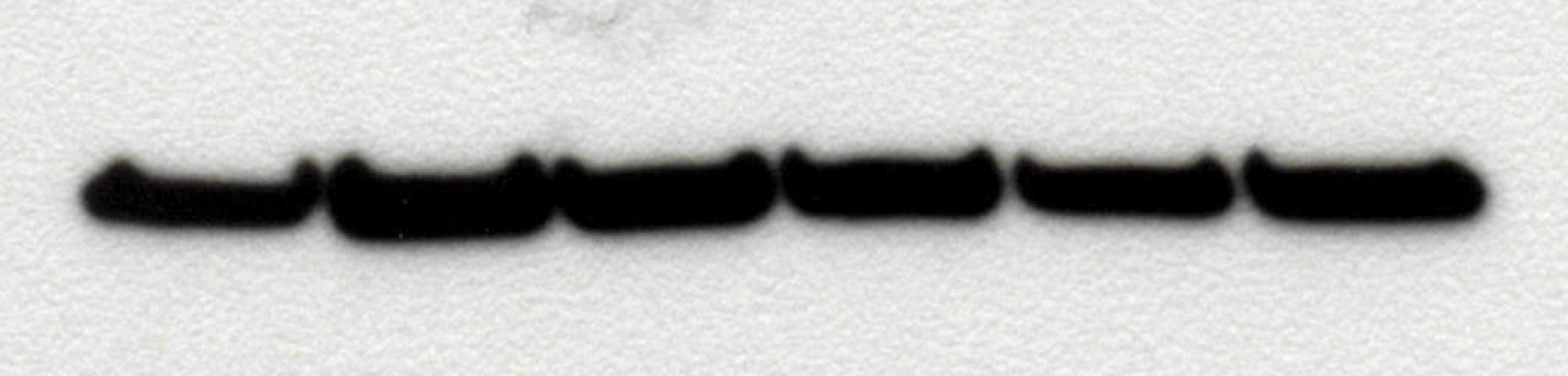

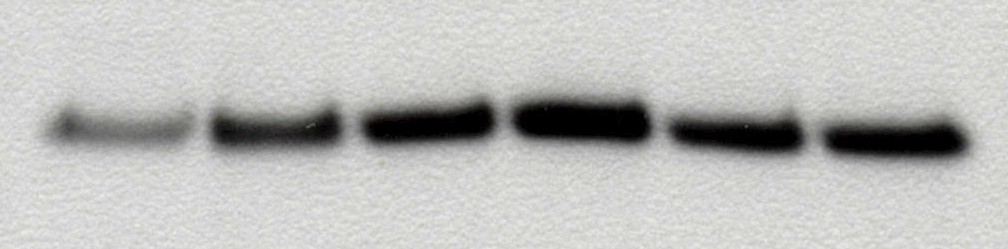

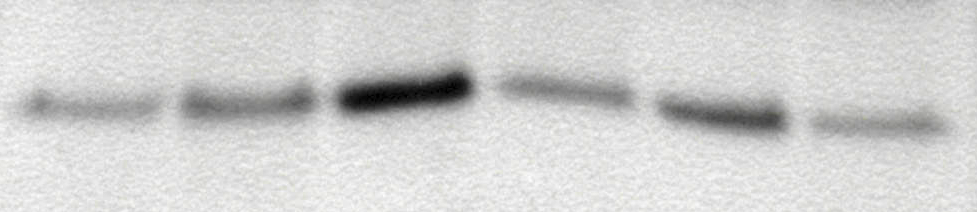


BCL-XL

BCL-W

ß-ACTIN


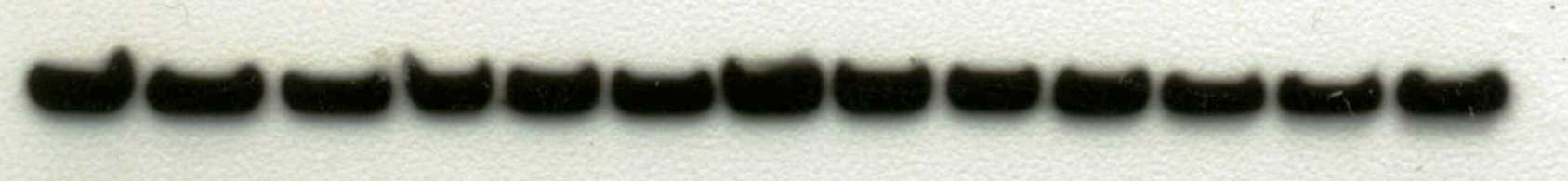

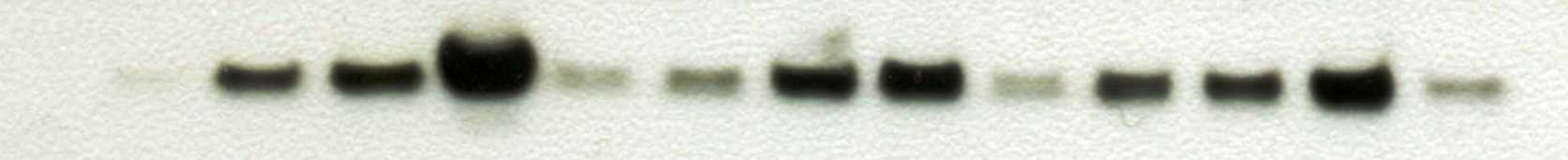

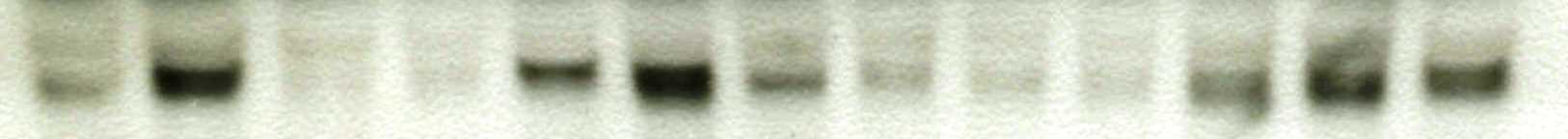


BCL-2

MCL-1

α-TUBULIN


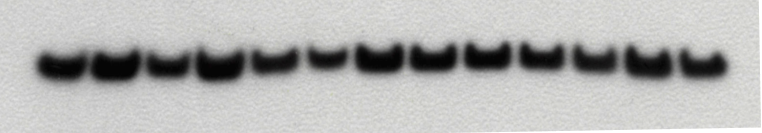

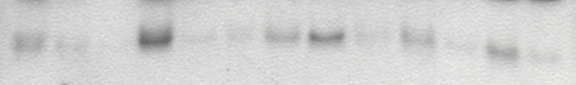

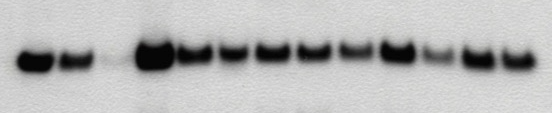


BCL-XL

BCL-W

ß-ACTIN

PDX6

PDX22

PDX1

PDX2

PDX13

PDX23

PDX8

PDX18

PDX5

PDX7

PDX10

PDX4

PDX15

**Supplementary Figure 6: Cytochrome c-release upon exposure of ALL cells to pro-apoptotic peptides**

Principle of BH3-profiling exemplified for the BIM peptide. ALL cells were permeabilized and exposed to increasing concentrations of the pro-apoptotic BH3 peptide BIM. Release of cytochrome c in ALL cells was analyzed using an anti-cytochrome c antibody that exclusively binds to cytochrome c retained in the mitochondria.


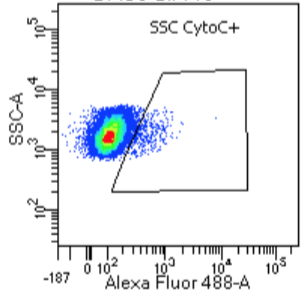

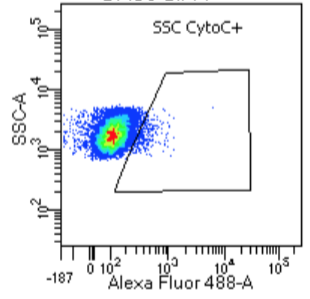

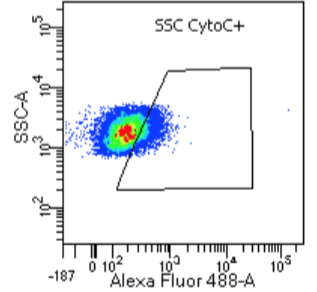

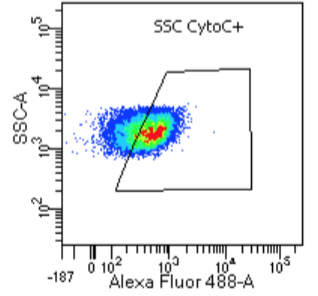

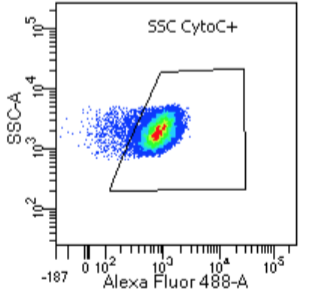


BIM 0.001 µM

BIM 0.01 µM

BIM 0.1 µM

BIM 1 µM

BIM 10 µM

mitochondrial cytochrome c

**Supplementary Figure 7: Association of mitochondrial profiling with *ex vivo* sensitivity to venetoclax**

Association (linear regression) of EC_50_ of venetoclax with the BH3 profiling parameter indicated. N=20 patient-derived xenograft BCP-ALL. R^2^, correlation coefficient. P, significance, Bonferroni-adjusted significance level: P ≤ 0.05/6 = P ≤ 0.0083. See also Table 2.

PUMA

cytochrome c release [%]

HRK

cytochrome c release [%]

log EC_50_ (nM Venetoclax)

R^2^=0.212, P=0.0413

R^2^=0.140, P=0.1039

BIM

cytochrome c release [%]

R^2^=0.203, P=0.0465

R^2^=0.051, P=0.3404

MS1

cytochrome c release [%]

**Supplementary Figure 8: *In vivo* venetoclax response and potential markers for venetoclax sensitivity**

Association (linear regression) of *in vivo* venetoclax response (‘delta survival’) with the parameter indicated. N=12 *in vivo* venetoclax treated patient-derived xenograft BCP-ALL. R^2^, correlation coefficient. P, significance, Bonferroni-adjusted significance level: P ≤ 0.05/4 = P ≤ 0.0125. See also Table 3.

log EC_50_

(nM Venetoclax)

BCL-2 / MCL-1

(protein ratio)

Delta survival [Days]

R^2^=0.311, P=0.0597

*BCL2*

(transcript)

R^2^=0.337, P=0.0479

R^2^=0.180, P=0.1698

*BCL2 / MCL1*

(transcript ratio)

R^2^=0.351, P=0.0423

**Supplementary Figure 9: Association of BAD-HRK with direct VEN priming**

Association (linear regression) of the parameter BAD-HRK with direct VEN priming. N=20 patient-derived xenograft BCP-ALL. R^2^, correlation coefficient. P, significance.

BAD-HRK

cytochrome c release [%]

VEN

cytochrome c release [%]

R^2^=0.606, P<0.0001

**Supplementary References:**

1 Drexler, H. G. Guide to Leukemia- Lymphoma Cell Lines, 2nd Edition. *Braunschweig* (2010).

2 Stong, R. C., Korsmeyer, S. J., Parkin, J. L., Arthur, D. C. & Kersey, J. H. Human acute leukemia cell line with the t(4;11) chromosomal rearrangement exhibits B lineage and monocytic characteristics. *Blood* **65**, 21-31 (1985).

3 Kim, D. H. *et al.* TEL-AML1 translocations with TEL and CDKN2 inactivation in acute lymphoblastic leukemia cell lines. *Blood* **88**, 785-794 (1996).

4 Duque-Afonso, J. *et al.* E2A-PBX1 Remodels Oncogenic Signaling Networks in B-cell Precursor Acute Lymphoid Leukemia. *Cancer research* **76**, 6937-6949, doi:10.1158/0008-5472.CAN-16-1899 (2016).

5 Wlodarska, I. *et al.* A new subtype of pre-B acute lymphoblastic leukemia with t(5;12)(q31q33;p12), molecularly and cytogenetically distinct from t(5;12) in chronic myelomonocytic leukemia. *Blood* **89**, 1716-1722 (1997).
